# Draft Genome Sequence of a Polymyxin B-Resistant Sequence Type 195 Clinical Isolate of *Acinetobacter baumannii* from India

**DOI:** 10.1128/genomeA.00031-18

**Published:** 2018-02-08

**Authors:** Bina Agarwal, Niraj Agarwala, Sarangapani Saikia, Snigdha Sarkar, Giasuddin Ahmed

**Affiliations:** a Department of Biotechnology, Gauhati University, Jalukbari, Assam, India; b Department of Botany, Gauhati University, Jalukbari, Assam, India; c IBT Hub, Rangia College, Rangia, Assam, India

## Abstract

*Acinetobacter baumannii* has emerged as a troublesome nosocomial pathogen worldwide. We report here the draft genome sequence of polymyxin B-resistant sequence type 195 (ST195) *A. baumannii* strain GU71, isolated from a tertiary care hospital in the city of Guwahati, Assam, India.

## GENOME ANNOUNCEMENT

*Acinetobacter baumannii* is a tenacious pathogen with a wide spectrum of antimicrobial resistance that causes nosocomial infections, mainly in immunocompromised patients (1). Recent reports showed that *A. baumannii* has become a major pathogen in Indian health care organizations (2, 3). Due to the emergence of carbapenem-resistant *A. baumannii* (CRAB) strains, polymyxins and tigecyclines are the only last-resort drugs available to treat infections caused by *A. baumannii*. This pathogen is rapidly developing resistance to polymyxins, such as polymyxin B and colistin. In this report, the draft genome sequence of a polymyxin B-resistant sequence type 195 (ST195) clinical isolate of *A. baumannii*, the GU71 strain, is discussed.

The strain sequenced in this report and designated GU71 was recovered from a blood sample of a 28-year-old female patient hospitalized in the city of Guwahati, Assam, India, in 2013. The GU71 strain was found to be resistant to a series of all antibiotics, except tigecycline and colistin, tested according to Clinical and Standards Institute (CLSI) guidelines. This extensively drug-resistant (XDR) strain was susceptible to colistin, but interestingly, it also showed resistance to another type of polymyxin, polymyxin B.

For isolation of genomic DNA, *A. baumannii* GU71 was grown overnight at 37°C on a Mueller-Hinton agar plate, which was followed by culturing in LB broth. Genomic DNA was extracted using a HiPurA bacterial genomic DNA purification kit according to the manufacturer’s instructions. The NEBNext Ultra DNA library preparation kit was used to construct the library for genome sequencing. The library was sequenced using an Illumina HiSeq 2500 platform generating 2 × 150-bp paired end reads, and a total of 5.2 million high-quality reads were obtained. The reads were assembled using Velvet assembler v1.2.10 (4). The resulting assembly consisted of 56 contigs comprising a total length of 3,968,977 bases, with an *N*_50_ contig size of 162,485 and a G+C content of 38.83%. Annotation of the draft assembly was performed using the NCBI Prokaryotic Genome Annotation Pipeline (PGAP), which predicted 3,884 genes, including 3,733 coding genes, 64 RNA genes (57 tRNAs and 4 non-coding RNAs [ncRNAs]), and 87 pseudogenes. ResFinder 3.0 (5) identified a total of 10 resistance-related genes, including three aminoglycoside genes (*strA*, *strB*, and *armA*), three β-lactam genes (*bla*_ADC-25_, *bla*_OXA-23_, and *bla*_OXA-66_), two macrolide genes [*msr*(E) and *mph*(E)], and one each of sulfonamide (*sul2*) and tetracycline resistance genes [*tet*(B)]. The presence of putative secondary metabolite gene clusters, including those encoding for a bacterial pigment (aryl polyene), an antibiotic (kijanimicin), siderophores (acinetoferrin and acinetobactin), and a bioemulsifier (emulsan), were predicted using antiSMASH tools (6). *In silico* multilocus sequence typing (MLST) analysis by pubmlst (https://pubmlst.org/abaumannii/) (7) assigned GU71 to ST195 and ST2 according to the Oxford and Pasteur schemes, respectively. This is the first draft genome sequence report for a polymyxin B-resistant *A. baumannii* ST195 strain from India. Genome annotation revealed the presence of the *pmrCAB* operon, which is known to facilitate polymyxin resistance in *A. baumannii* (8). Comparative genomics study involving GU71 can help to uncover the polymyxin B resistance mechanism in this bacterium, which largely remains unknown. In summary, the draft genome of this clinical *A. baumannii* GU71 strain will further speed up our understanding of acquisition and spread of antibiotic resistance in this highly pathogenic bacterium. Additionally, the genome sequence can serve as a valuable resource for study of the evolutionary dynamics of *A. baumannii*.

### Accession number(s).

This whole-genome shotgun project has been deposited at DDBJ/EMBL/GenBank under the accession number PEIF00000000. The version described in this paper is the first version, PEIF01000000.
